# The Coiled Coils of Cohesin Are Conserved in Animals, but Not In Yeast

**DOI:** 10.1371/journal.pone.0004674

**Published:** 2009-03-05

**Authors:** Glenn E. White, Harold P. Erickson

**Affiliations:** 1 Department of Biological and Environmental Sciences, Longwood University, Farmville, Virginia, United Kingdom; 2 Department of Cell Biology, Duke University Medical Center, Durham, North Carolina, United States of America; German Cancer Research Center, Germany

## Abstract

**Background:**

The SMC proteins are involved in DNA repair, chromosome condensation, and sister chromatid cohesion throughout Eukaryota. Long, anti-parallel coiled coils are a prominent feature of SMC proteins, and are thought to serve as spacer rods to provide an elongated structure and to separate domains. We reported recently that the coiled coils of mammalian condensin (SMC2/4) showed moderate sequence divergence (≈10–15%) consistent with their functioning as spacer rods. The coiled coils of mammalian cohesins (SMC1/3), however, were very highly constrained, with amino acid sequence divergence typically <0.5%. These coiled coils are among the most highly conserved mammalian proteins, suggesting that they make extensive contacts over their entire surface.

**Methodology/Principal Findings:**

Here, we broaden our initial analysis of condensin and cohesin to include additional vertebrate and invertebrate organisms and multiple species of yeast. We found that the coiled coils of SMC1/3 are highly constrained in *Drosophila* and other insects, and more generally across all animal species. However, in yeast they are no more constrained than the coils of SMC2/4 and Ndc80/Nuf2p, suggesting that they are serving primarily as spacer rods.

**Conclusions/Significance:**

SMC1/3 functions for sister chromatid cohesion in all species. Since its coiled coils apparently serve only as spacer rods in yeast, it is likely that this is sufficient for sister chromatid cohesion in all species. This suggests an additional function in animals that constrains the sequence of the coiled coils. Several recent studies have demonstrated that cohesin has a role in gene expression in post-mitotic neurons of *Drosophila*, and other animal cells. Some variants of human Cornelia de Lange Syndrome involve mutations in human SMC1/3. We suggest that the role of cohesin in gene expression may involve intimate contact of the coiled coils of SMC1/3, and impose the constraint on sequence divergence.

## Introduction

**Figure 1:**
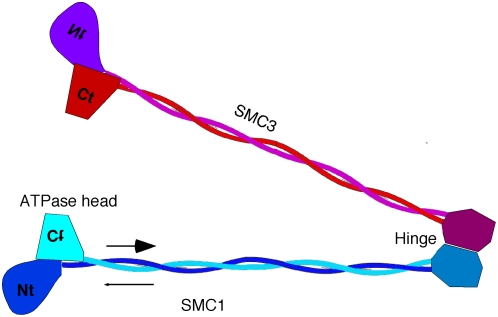
SMC protein structure. SMC proteins have a hinge region in the middle of the sequence where the protein folds to form long segments of anti-parallel coiled coils. This brings the N-terminus (Nt) and C-terminus (Ct) together to form the ATPase heads. The hinge region of each protein interacts to form the heterodimer. The head of SMC1 can bind the head of SMC3 to make a ring. The linked heads are stabilized by binding of accessory proteins (not shown). The coiled coils are drawn to scale.

The structural maintenance of chromosome (SMC) proteins interact with DNA to carry out several critical functions within the cell including DNA repair, chromosome condensation, and sister chromatid cohesion during mitosis [1]–[8]. Three SMC protein complexes have been identified in eukaryotes: SMC1/3 (cohesin), SMC2/4 (condensin), and SMC5/6. Each SMC complex consists of two SMC protein subunits and a varying number of accessory proteins. The SMCs are structurally characterized by a long, anti-parallel coiled coil that exhibits the hydrophobic packing of Crick [9]. The SMC subunits fold at the central hinge, and the coiled coils bring the N- and C-terminal domains together to form the ATPase head domain (Fig. 1). The two SMC subunits of each pair form a dimer, with the hinges associated in the middle and the ATPase domains at the ends of the long coiled-coil rods (Fig. 1).

Research efforts on the function of cohesin (SMC1/3 and two associated proteins) have focused primarily on its well-documented role in holding sister chromatids together until they are ready for separation in anaphase [10]–[15], and recently to a role in mitotic spindle pole formation [16]. During mitosis, SMC1 complexes with SMC3 to form cohesin (we use SMC1 to designate SMC1p (yeast) and SMC1A or SMC1L1 (animals)). During meiosis in animals, a second isoform (SMC1β) complexes with SMC3 to form a second cohesin [17], [18]. An attractive model for the mechanism of sister chromatid cohesion proposes that the ATPase heads come together and the long coiled coils form a ring that could trap the DNA of each sister chromatid ([5], [19], but see also [20]). In this model, and indeed in most thinking about the possible function of cohesin and condensin, the coiled coils are thought to serve a structural role as long spacer arms. However, we recently reported that the mammalian cohesins possess the most highly conserved coiled-coil domains in the genome [21], suggesting that they are not just spacer arms (discussed below).

Emerging evidence demonstrates that cohesin also has important functions in interphase cells, independent of its role in cohesion. Cohesin binds to specific chromosomal sites across the genome, and is involved in regulating gene expression in post-mitotic cells. In yeast, the cleavage of the Scc1/Mdc1p subunits of cohesin play a role in transcriptional silencing [22] and SMC1 and SMC3 may be a part of the mechanism defining the boundaries of the transcriptionally silenced HMR locus [23]. Cohesin mutations in the coiled coils of both the SMC1 and SMC3 subunits were discovered in patients with a mild variant of Cornelia de Lange Syndrome (CdLS) [24]–[27] – a human neurological developmental disorder associated with mental retardation. In *Drosophila*, studies with cohesin mutants demonstrated that cohesin is required for developmental axonal pruning [28] and abnormal cohesin complex cleavage alters wild type larval locomotion [29]. Finally, cohesin binding sites and those of the zinc-finger protein CCCTC-binding factor (CTCF), which acts as an insulator protein that blocks enhancer-promoter interactions, overlap significantly in mammalian chromosomes ([30]–[34]; see [33], [34] for commentary). CTCF is found in *Drosophila* and vertebrates [35]. 9,000 cohesin-binding sites were found in the human genome, and 90% of them also bound CTCF [31]–[33]. Additionally, this post-mitotic function for cohesin appears not to be limited to neurons as immunoblots demonstrate that cohesin is differentially expressed in a variety of murine tissue extracts [32]. Overall, these studies demonstrate that cohesin functions in regulating gene expression in addition to its role in sister chromatid cohesion.

In a previous study, we noted that the coiled-coil segments of SMC1 and SMC3 were among the most highly conserved mammalian proteins, showing sequence divergence across different mammalian species of only 0–1% over ∼700 amino acids [21]. To put this in context, we analyzed a variety of coiled-coil proteins. Some coiled coils (Ndc80, Nuf2p, giantin) showed sequence divergence of ∼20% across different species of mammals, and we concluded that this was a typical sequence divergence of coiled coils serving primarily as inert spacer rods. Ndc80/Nuf2p appear to be found in all eukaryotes and form a heterodimeric coiled coil that is involved in linking the kinetochore to microtubules [36]–[38]. The coiled coils of condensins (SMC2/4) showed 10–15% sequence divergence, suggesting that they are also serving primarily as rods. Proteins that are known to use their coiled coils for packing into filaments, such as skeletal muscle myosin II and intermediate filament proteins, showed a divergence of only 1–3%, reflecting the constraints to maintain the protein-protein contacts over their surface. The coiled coils of SMC1/3 were even more highly conserved than myosin II and intermediate filament proteins, which implied these coiled coils are not serving just as spacer rods. We concluded that the coiled coils of SMC1/3 probably have interactions over their entire length and circumference that constrain sequence divergence.

We also found that the coiled coils of SMC1/3 were much more highly conserved than those of SMC2/4 when we compared human sequences to avian, amphibian, and *Drosophila* orthologs [21]. This suggested that the mechanism imposing the constraint in SMC1/3 coiled coils was found in all vertebrates, and perhaps all animal species, and raised the question whether this mechanism is found in all eukaryotes. As genomic sequences have become increasingly available for a number of yeast, nematode, *Drosophila*, and other invertebrate species [39]–[43], we decided to extend our analysis to yeast and additional animal species. We determined that the coiled coils of SMC1/3 are more conserved than those of SMC2/4 and Ndc80/Nuf2p in vertebrates and several species of invertebrates, but are no more conserved than those of SMC2/4 and Ndc80/Nuf2p across multiple yeast species. In addition, the meiotic SMC1β coils are not constrained in animals.

Tropomyosin, an actin binding coiled-coil protein found throughout Eukaryota served as our control reference sequence of a coiled coil that is universally highly conserved [9], [44], [45]. Tropomyosin is an essential element of the thin filament of striated muscle while its non-muscle isoforms have been implicated in a number of functions including actin filament stability and cytokinesis [46], [47]. Its sequence conservation is apparently due to its binding to actin and the troponin proteins, involving most of the surface of the coiled coil. Our finding that SMC1/3 coils are not constrained in yeast suggests that there is a special mechanism involving the SMC1/3 coils that operates in metazoans but not in yeast nor in animal cells undergoing meiosis.

## Methods

### Protein Sequence Acquisition and Coiled-Coil Domain Determination

Seven coiled-coil proteins (SMC1/3, SMC2/4, Ndc80, Nuf2p, tropomyosin (Tm)) from *Saccharomyces cerevisiae*, *Caenorhabditis elegans*, *Drosophila melanogaster*, and *Homo sapiens* served as the reference sequences for this study. Protein sequences for each were obtained from GenBank with the majority annotated as Reference Sequences. The remaining sequences were obtained by using these sequences as the query in either a BLAST search of GenBank's protein database, or a Eukaryota Genomic BLAST (tBLASTn) search of the whole genome shotgun sequence databases (http://www.ncbi.nlm.nih.gov/sutils/genom_table.cgi?organismeuk). For those species which only had whole genome shotgun sequences available, orthologous proteins were identified based upon the highest % identity with the query sequence for that organism, and that their coiled-coiled domains, as identified using the COILS prediction program (see below), closely approximated those of the query sequences. The gene sequences were then translated using the SIXFRAME tool at Biology Workbench at the San Diego Super Computer (http://seqtool.sdsc.edu/CGI/BW.cgi).

The coiled-coil domains of each protein were identified using the 28-residue window output from the COILS program (http://www.ch.embnet.org/software/COILS_form.html) and to maintain consistency with our previous study, only those regions of the sequence where the score was ≥0.5 were used for the analysis of the coils [21], [48]. All SMC proteins in this study had disruptions of their coiled coils [49]. The boundaries of the individual coiled-coil segments were identified and the non-coil segments were removed. For a select subset of SMC proteins, coiled coil boundaries were determined using other coiled coil prediction software including PairCoil2 ([50]; http://groups.csail.mit.edu/cb/paircoil2/) and PCOILS ([51]; http://toolkit.tuebingen.mpg.de/pcoils). All three programs predicted slightly different boundaries for the coiled-coil segments, and using our analysis criteria, resulted in slightly different percent divergences. However, the sequence divergences never changed more than 1 or 2 percent, so the trends we report using the COILS program (in this and our previous study), are not affected by the small differences in boundaries.

For ease of viewing, the SMC1/3, SMC2/4, Ndc80, Nuf2p and tropomyosin sequences are grouped by organism. For those sequences that served as the reference sequences in this study, the following additional information is provided: 1) protein length (or noted as a partial sequence), and 2) the boundaries of the coiled-coil segments as determined by COILS. All remaining sequences are listed by their GenBank accession number only with translated ORFs and partial length sequences reported as such. In some instances, the protein sequence was reconstructed from multiple genomic segments, and that is noted.

As all sequences (except for those from *Ciona intestinalis*) were obtained from GenBank, only their accession numbers are provided.

### Yeast

*Saccharomyces cerevisiae* SMC1 ( = SMC1p) [NP_116647.1, 1225 amino acids, N and C Coil: G174 - T212, E232 - S283, Q320 - T387, Y395 - I441, S456 - L496, S707 - Y747, E753 - K795, N805 - N943, K987 - E1065], SMC3 [NP_012461.1, 1230 amino acids, N and C Coil: A172 - Y230, S259 - Q347, R358 - K387, I403 - G451, K455 - L482, R484 - M511, L677 - L737, R744 - S842, D860 - I957, D971 - D1042], SMC2 [NP_116687.1, 1170 amino acids, N and C Coil: K172 - E204, S248 - T386, G398 - E435, V678 - Q712, Q742 - F946, V953 - N983, N985 - E1027], SMC4 [NP_013187, 1418 amino acids, N and C Coil: M344 - G374, K399 - L676, E849 - G971, D1008 - S1118, V1224 - R1263], Ndc80 [NP_012122.1, 691 amino acids, N and C Coil: I315 - N362, K368 - E452, I521 - Q645, K655 - N688], Nuf2p [NP_014572.1, 451 amino acids, N and C Coil: L145 - Q221, S248 - L297, I340 - L376, Q399 - Q451], and Tm [CAA60179.1, 199 amino acids, Coil: M1 - L199 (entire protein)].

*Saccharomyces bayanus* SMC1 [AACG02000045, translated ORF], SMC3 [AACG02000110, translated ORF], SMC2 [AACG02000107, translated ORF], SMC4 [AACG02000004.1, translated ORF], Ndc80 [AACG02000025.1, translated ORF], Nuf2p [AACG02000237.1, translated ORF, (partial)], and Tm [AACG02000029.1, translated ORF].

*Saccharomyces castellii* SMC1 [ACF01000015, translated ORF], SMC3 [AACF01000014.1, translated ORF], SMC2 [AACF01000153.1, translated ORF], SMC4 [AACF01000036.1, translated ORF], Ndc80 [AACF01000021.1, translated ORF, (partial)], Nuf2p [AACF01000007.1, translated ORF], and Tm [AACF01000030.1, translated ORF].

*Saccharomyces kluyveri* SMC1 [AACE02000190, translated ORF], SMC3 [AACE02000434.1], SMC2 [AACE02000042, translated ORF], SMC4 [Not available], and Ndc80 [AACE03000003.1, translated ORF, (partial)], Nuf2p [AACE03000005.1, translated ORF (partial)], and Tm [AACE03000003.1, translated ORF, (partial)].

*Saccharomyces kudriavzevii* SMC1 [AACI02000104, translated ORF], SMC3 [Not available], SMC 2 [Not available], SMC4 [AACI02000158.1, translated ORF], Ndc80 [AACI02000184.1, translated ORF, (partial)], Nuf2p [AACI02000751.1, translated ORF], and Tm [AACI02000420.1, translated ORF].

*Saccharomyces mikatae* SMC1 [AABZ01000094, translated ORF], SMC3 [AABZ01000321.1 and AABZ01000532.1, reconstructed protein sequence from translated ORF], SMC2 [AABZ01000019.1, translated ORF], SMC4 [Not available], Ndc80 [AABZ01000015.1, translated ORF], Nuf2p [AACH01000844.1, translated ORF], and Tm [AACH01000004.1, translated ORF].

*Saccharomyces paradoxus* SMC1 [AABY01000015.1, translated ORF], SMC3 [AABY01000267.1, translated ORF], SMC2 [AABY01000021.1, translated ORF], SMC4 [AABY01000055.1, translated ORF], Ndc80 [AABY01000004.1, translated ORF], Nuf2p [AABY01000044.1, translated ORF], and Tm [AABY01000091.1, translated ORF, (partial)].

*Ashbya gossypii* ATCC 10895 SMC1 [NP_986643.1], SMC3 [NP_982360.1], SMC2 [NP_986902], SMC4 [NP_986754], Ndc80 [Not available], Nuf2p [NP_984846.1], and Tm [NC_005786.1 (partial)].

*Candida glabrata* SMC1 [XP_446063.1], SMC3 [XP_446916.1], SMC2 [XP_445638.1], SMC4 [XP_449298.1], Ndc80 [XP_445532.1], Nuf2p [XP_448216.1], and Tm [NC_006033.1].

*Kluyveromyces lactis* SMC1 [XP_453397.1], SMC3 [XP_451012.1], SMC2 [XP_453765], SMC4 [XP_455936.1], Ndc80 [XP_454536.1 (partial)], Nuf2p [XP_456083.1], and Tm [NC_006041.1 (partial)].

*Kluyveromyces waltii* SMC1 [AADM01000020, translated ORF], SMC3 [AADM01000325.1, translated ORF], SMC2 [AADM01000229.1, translated ORF], SMC4 [AADM01000282.1, translated ORF], Ndc80 [AADM01000214.1, translated ORF, (partial)], Nuf2p [AADM01000270.1, translated ORF, (partial)], and Tm [AADM01000003.1, translated ORF (partial)].

### Nematode

*Caenorhabditis elegans* SMC1 [NP_001040658.1, 1281 amino acids, N and C Coil: T164 - L235, H238 - Q408, L417 - A489, A673 - L760, R763 - F793, N810 - L933, N1043 - L1108], SMC3 [NP_499453.2, 1261 amino acids, L180 - V334, K394 - F452, Q471 - A503, D675 - F750, E758 - L836, K842 - L947, R1023 - A1088], SMC2 [NP_496331.1, 1244 amino acids, N and C Coil: C244 - L303, Q306 - K355, I415 - E479, Q702 - E954, S1004 - E1044], SMC4 [NP_497935.1, 1549 amino acids, N and C Coil: M326 -T490, A492 - K604, K786 - S832, A844 - R1058, E1144 - R1213], Ndc80 [NP_501830.1, 590 amino acids, N and C Coil: C262 - N296, H298 - H342, K347 - E376, R442 - I471, R488 - D525, S527 - A555], Nuf2p [Q21952.1, 490 amino acids, N and C Coil: A136 - V242, E247 - I283, E303 - N412], and Tm [NP_001021698.1, 284 amino acids, Coil: M1 - V284 (all)].

*Caenorhabditis briggsae* SMC1 [CAE73105.1 (partial)], SMC3 [XP_001664944.1], SMC2 [XP_001679291.1], SMC4 [XP_001665682.1], Ndc80 [CAP39778.1, (partial)], Nuf2p [XP_001664731.1], and Tm [CAQ00070.1].

*Brugia malayi* SMC1 [EDP37102.1 (partial)], SMC3 [EDP31459.1], SMC2 [EDP30866.1], SMC4 [Not available], Ndc80 [XP_001894611.1], Nuf2p [XP_001895760.1 (partial)], and Tm [AAQA01004217.1, NZ_AAQA01003177.1, NZ_AAQA01001627.1, reconstructed protein sequence from translated ORF, (partial)].

### Insect

*Drosophila melanogaster* SMC1 [CAB76376.1, 1238 amino acids, N and C Coil: L186 - Q247, H253 - R318, L320 - V526, H683 - S804, V811 - N951, N1037 - R1082], SMC3 [NP_523374.2, 1200 amino acids, N and C Coil: E173 - Q379, N397 - A501, N671 - D730, K756 - N834, A846 - A948, R958 - F989], SMC2 [NP_610995.1, 1179 amino acids, N and C Coil: Q173 - E201, K255 - M292, T312 - Q385, T389 - Q432, D441 - Q507, A670 - L724, M726 - K782, R785 - I939, V988 - I1017], SMC4 [NP_723996.1, 1409 amino acids, N and C Coil: L272 - F307, I328 - S592, A777 - K1020], Ndc80 [NP_572902.1, 675 amino acids, N and C Coil: K302 - L429, D525 - D559, R565 - L662] , Nuf2p [AAC05669.1, 502 amino acids, Coil: E205 - R383, T388 - T432, L449 - I486] and Tm [NP_732012.1, 284 amino acids, Coil: M1 - Y284 (all)].

*Drosophila sechellia* SMC1 [AAKO01000393.1, translated ORF], SMC3 [AAKO01002722.1 and AAKO01000647.1, reconstructed protein sequence from translated ORF], SMC2 [AAKO01000647.1, reconstructed protein sequence from translated ORF], SMC4 [AAKO01001629.1, translated ORF, (partial)], Ndc80 [AAKO01002874.1, translated ORF, (partial)], Nuf2p [AAKO01000125.1 (partial)], and Tm [XP_002031091.1].

*Drosophila pseudoobscura* SMC1 [XP_001358804.1], SMC3 [EAL31729.1 (partial)], SMC2 [NZ_AADE01002186.1, reconstructed protein sequence from translated ORF], SMC4 [XP_001356541.1], Ndc80 [XP_001354724.1], Nuf2p [XP_001353684.1], and Tm [XP_001359903.2].

*Drosophila grimshawi* SMC1 [AAPT01019331, translated ORF, reconstructed protein sequence], SMC3 [AAPT01020284.1, translated ORF], SMC2 [AAPT01021513.1, translated ORF, reconstructed protein sequence], SMC4 [AAPT01021775.1, translated ORF, reconstructed protein sequence (partial)], Ndc80 [AAPT01021199.1, translated ORF, (partial)], Nuf2p [AAPT01020686.1, translated ORF, (partial)], and Tm [XP_001989542.1].

*Anopheles gambiae* SMC1 [XP_311953.3], SMC3 [XP_316422.3], SMC2 [XP_554796.1], SMC4 [XP_317674.2], Ndc80 [XP_001688950.1], Nuf2p [EAA07182.5], and Tm [XP_321262.1].

*Aedes aegypti* SMC1 [XP_001651465.1], SMC3 [XP_001652359.1], SMC2 [XP_001656789.1], SMC4 [XP_001653748.1], Ndc80 [XP_001661493.1], Nuf2p [Not available], and Tm [XP_001655948.1].

*Apis mellifera* SMC1 [XP_395059.2], SMC3 [XP_393700.2], SMC2 [XP_396284.2], SMC4 [XP_396862.3], Ndc80 [Not available], Nuf2p [Not available], and Tm [XP_391961.2].

### Additional Animal Sequences

*Homo sapiens* SMC1 ( = SMC1L1 = SMC1A) [NP_006297.2, 1233 amino acids, N and C Coil: A178 - I503, D658 - T724, P746 - R782, V786 - Q936, L990 - D1068], SMC3 [NP_005436.1, 1217 amino acids, N and C Coil: E173 - K215, R220 - R360, S387 - L472, R474 - A504, G667 - G947, T959 - E1022], SMC2 [CAD89875.1, 1197 amino acids, N and C Coil: R172 - T204, Q238 - L362, A396 - R507, A671 - A884, I889 - D937, R981 - N1031], SMC4 [NP_001002800.1, 1288 amino acids, N and C Coil: R272 - A305, Y326 - S588, L767 - E1134], SMC1β [NP_683515.3, 1235 amino acids, N and C Coil: E156 - F226, L256 - N304, S306 - D490, W666 - K725, Q736 - E782, V786 - Q880, S883 - K934, D967 - E994, R1022 - R1049], Ndc80p [NP_006092.1, 642 amino acids, N and C Coil: K253 - K423, Q456 - A548, L550 - K557, A605 - E642], Nuf2p [NP_663735.1, 464 amino acids, N and C Coil: L142 - V172, Q211 - D284, S288 - Q356, V387 - R457], and Tm [NP_001018005.1, 284 amino acids, Coil: M1 - I284 (all), alpha chain isoform 1].

*Bos taurus* SMC1 [NP_7770039.1], SMC3 [NP_776720.1], SMC2 [Not available], SMC4 [Not available], SMC1β [XP_600396.3, (partial)], Ndc80 [XP_582722.3], Nuf2p [NP_001092481.1], and Tm [NP_001013608.1].

*Mus musculus* SMC1 [NP_062684.1], SMC3 [NP_031816.2], SMC2 [NP_032043.2], SMC4 [NP_598547.1], SMC1β [NP_536718.1, Ndc80p [NP_075783.1], Nuf2p [NP_075773.1], and Tm [P58771.1]

*Rattus norvegicus* SMC1 [NP_113871.1], SMC3 [NP_113771.1], SMC2 [XP_342838.1], SMC4 [XP_215573.2], SMC1β [XP_217011.3], Ndc80p [XP_217489.3], Nuf2p [NP_001012028.1], and Tm [NP_001029240.1].

*Gallus gallus* SMC1 [NP_989847.1], SMC3 [NP_989848.1], SMC2 [NP_990561.1], SMC4 [NP_989849.1], SMC1β [XP_416467.2], Ndc80 [NP_989808.2], Nuf2p [Q76I90], and Tm [P04268.2].

*Xenopus laevis* SMC1 [NP_001080490], SMC3 [CAD59446.1], SMC2 [P50533], SMC4 [P50532], SMC1β [Not available], Ndc80 [NP_001082372.1], Nuf2p [NP_001082370.1], and Tm [NP_001128548.1].

*Danio rerio* SMC1 [XP_688120], SMC3 [NP_999854.1], SMC2 [XP_001918836.1], SMC4 [NP_775360.1], SMC1β [XP_001334257.1], Ndc80 [NP_001003863.1], Nuf2p [NP_956604.1], and Tm [NP_571180.1].

*Strongylocentrotus purpuratus* SMC1 [XP_001177541.1], SMC3 [XP_001202053.1 and XP_001176817, reconstructed protein sequence, (partial)], SMC2 [Not available], SMC4 (XCAP-C) [XP_797583.2], Ndc80 [XP_001197919.1], Nuf2p [Not available], and Tm [XP_001202479.1, (partial)].

*Ciona intestinalis* SMC1 [Ciona:290090], SMC3 [Ciona:236566], SMC2 [Ciona:269866], SMC4 [Ciona:259758, (partial)], Ndc80 , Nuf2p [Not available], and Tm [Not available]. Sequences were downloaded from the *Ciona intestinalis* v2.0 website: http://genome.jgi-psf.org/Cioin2/Cioin2.home.html.

*Nematostella vectensis* SMC1 [XP_001641659], SMC3 [XP_001626236], SMC2 [Not available], SMC4 [XP_001632709], Ndc80 [XP_001630759], Nuf2p [Not available], and Tm [XP_001629293.1].

### Amino Acid Sequence Divergence

After identification of the coiled-coil domains for each protein, all non-coil segments were removed from the sequence. The N- and C-terminal coiled coils were then combined and analyzed as one continuous coil. Orthologs were always compared to the reference sequence for each organism. A preliminary alignment of the proteins was used to identify sequence gaps that were removed prior to the determination of sequence divergence. Each pair of sequences was aligned using the “BLAST 2 sequences” tool accessed through the Biology WorkBench of the San Diego Super Computer (http://seqtool.sdsc.edu/CGI/BW.cgi). The percent amino acid sequence divergence was calculated from the output of the paired alignment. As with previous work [21], we used the simplest measure of sequence divergence, i.e., the percent of amino acid changes between the two sequences. Conserved amino acid substitutions were not considered.

## Results

We analyzed the sequence divergence of the coiled-coil domains of Tm, Ndc80/Nuf2p, SMC2/4, and SMC1/3 in multiple species of yeast, nematodes, insects, and other animals. Overall, the most important comparison is the sequence divergence of the coiled coils of SMC1/3 compared to SMC2/4, which we believe function primarily as spacer rods.

Table 1 shows the sequence divergence across 10 species of yeast, each compared to *S. cerevisiae*. The analysis is most informative for the first four, which are the closest to *S. cerevisiae* (members of *Saccharomyces sensu stricto* [40], and which diverged approximately 10-20 MYA (5.26K generations/year) [52], [53]), but the conclusions are similar for the more divergent species. The coils of SMC1/3 are no more conserved than the coils of SMC2/4 or Ndc80/Nuf2p. Our reference coiled-coil protein tropomyosin, however, is highly constrained across these same species, providing a benchmark coiled coil sequence within Saccharomycotina. The fact that tropomyosin's coils are highly constrained seems consistent with its recently demonstrated essential role in cytokinesis [47], and the involvement of much of its surface in binding to actin.

**Table 1:** Yeast (Saccharomycotina) Tropomyosin, Ndc80, Nuf2p, and SMC Protein and Coil Divergences.

% AA Divergence vs. *S. cerevisiae* *							
	Tm	Ndc80	Nuf2p	SMC2	SMC4	SMC1	SMC3
*S. paradoxus* *	1.6	4.5 (5.5)	3.7 (2.9)	7.3 (5.8)	11.3 (9.1)	5.4 (4.3)	6.8 (5.2)
*S. mikatae* *	1.5	10.0 (9.4)	9.6 (8.0)	17.7 (12.3)	NA	9.7 (7.5)	12.2 (8.7)
*S. bayanus* *	8.0	12.4 (11.8 p)	10.9 (8.5 p)	18.9 (12.3)	20.2 (17.4)	15.0 (9.8)	17.3 (10.0)
*S. kudriavzevii* *	3.5	11.7 (10.5 p)	11.4 (9.3)	NA	21.6 (17.9)	15.6 (18.0)	NA
*S. castellii*	35.7	57.0 (51.7)	57.2 (48.2)	49.4 (36.4)	54.8 (40.5)	44.9 (34.1)	44.2 (34.4)
*S. kluyveri*	25.2	59.4 (51.4)	47.0 (47.4 p)	50.1 (38.6)	NA	48.7 (41.1)	52.4 (43.2)
*C. glabrata*	24.6	61.9 (56.8)	61.6 (53.2)	54.3 (40.4)	67.3 (48.7)	61.7 (43.5)	42.4 (41.9)
*A. gossypii*	30.2	NA	56.7 (57.8)	51.6 (39.1)	61.9 (46.1)	57.3 (46.9)	56.1 (47.1)
*K. waltii*	26.3	59.9 (53.5)	57.2 (52.0)	57.9 (39.7)	58.8 (45.5)	60.1 (47.0)	60.1 (48.3)
*K. lactis*	32.3	66.3 (57.2)	58.6 (52.1)	56.4 (40.5)	64.6 (48.4)	59.0 (48.1)	62.0 (50.6)
*K. lactis vs. K. waltii*	19.5	65.8 (52.9)	54.7 (45.5)	58.3 (38.3)	63.8 (48.0)	65.8 (51.0)	56.9 (51.7)

Data presented as percentage sequence divergence for the combined coiled-coils (and in parentheses the divergence for the whole protein).

Tropomyosin sequences are 100% coiled-coil.

NA = sequence not available.

p = sequence is partial length (see Methods).

* *Saccharomyces sensu stricto*.

Table 2 shows the same comparisons for insects and nematodes. Based upon the *Drosophila* phylogeny recently generated from the analysis of whole genome shotgun sequences, three species of *Drosophila* were selected for comparison to *D. melanogaster*: *D. sechellia* (time to last common ancestor (TLCA)≈1.2 MYA (10 generations/year)), *D. pseudoobscura* (TLCA≈24 MYA), and *D. grimshawi* (TLCA≈40 MYA) [43], [54], [55]. In several insect species, the Ndc80/Nuf2p sequences returned from the tBLASTn search of the whole genome shotgun sequences showed either exceptionally high or low sequence divergence compared to *D. melanogaster*. We included in the tables all sequences that seemed to have the correct domain structure, in particular coiled coils of approximately the right length and position. We note that Ndc80 shows a very high divergence and Nuf2p a very low divergence across *Drosophila* species, but have not explored this further. Curiously, when other animal Ndc80/Nuf2p sequences are compared to Ndc80/Nuf2p from humans, it is the Ndc80 coil that is constrained relative to the Nuf2p [21]. The functional significance of these results is not clear at this time. Finally, tropomyosin coils are constrained in these insects but at different levels in *Drosophila* and mosquitoes.

**Table 2:** Nematode and Insect Tropomyosin, Ndc80, Nuf2p, and SMC Protein and Coil Divergences.

Insects							
% AA Divergence vs. *D. melanogaster*							
	Tm	Ndc80	Nuf2p	SMC2	SMC4	SMC1	SMC3
*D. sechellia*	4.6	6.8 (8.3 p)	1.3 (9.7)	3.8 (1.8)	3.4 (2.7)	0.3 (4.5)	0.2 (0.3)
*D. pseudoobscura*	6.3	63.4 (54.9)	8.7 (11.9)	27.3 (19.4)	32.2 (19.4)	6.4 (6.6)	4.3 (3.3)
*D. grimshawi*	6.0	64.0 (60.7p)	7.6 (23.5p)	31.3 (23.3)	37.9 (32.6)	8.2 (8.7)	6.8 (5.1)
*Anopheles gambiae*	21.1	73.2 (77.7)	31.9 (51.9)	58.4 (46.2)	62.0 (48.6)	34.8 (30.9)	28.1 (21.2)
*Aedes aegypti*	20.1	74.9 (78.6)	NA	58.0 (44.7)	61.2 (45.6)	33.1 (30.1)	27.2 (19.3)
*Apis mellifera*	17.3	NA	NA	63.8 (55.3)	73.8 (56.5)	60.4 (39.1)	57.1 (32.3)
*A. gambiae vs. A. aegypti*	4.6	67.8 (60.1)	NA	29.2 (22.8)	28.9 (24.0)	16.2 (15.5)	16.5 (10.6)

Nematodes							
% AA Divergence vs. *C. elegans*							
	Tm	Ndc80	Nuf2p	SMC2	SMC4	SMC1	SMC3
*C. briggsae*	7.0	44.4 (42.4)	45.5 (46.9)	59.8 (49.0)	47.5 (38.1)	32.4 (24.1)	37.3 (32.0)
*B. malayi*	11.8p	85.1 (75.6)	80.4 (80.1)	73.6 (65.1)	74.0 (69.3)	63.0 (50.7)	58.8 (52.5)

Data presented as percentage sequence divergence for the combined coiled-coils (and in parentheses the divergence for the whole protein).

Tropomyosin sequences are all coiled-coil.

NA = sequence not available.

p = sequence is partial (see Methods).

In contrast to yeast, insect species including *Drosophila* and mosquitoes (*Drosophila*/*Anopheles* TLCA≈250 MYA [56]) show a strong conservation of SMC1/3 coils relative to SMC2/4. This suggests that there is a mechanism constraining sequence divergence of cohesin's coiled coils, and this mechanism is found across all insect species. The nematode comparisons are less definitive but provide similar results. Their SMC1/3 coils are more conserved than are those of SMC2/4 and Ndc80/Nuf2p (*C. elegans/C. briggsae* TLCA≈24 MYA (6 generations/year in soil) [55]), but the difference is not as striking as in *Drosophila* and mammals, perhaps reflecting their lack of CTCF [35].

Table 3 extends our previous analysis of mammals and vertebrates to include more distant members of the animal kingdom. The conservation of SMC1/3 coils is especially striking for mammals (0-0.6% divergence), but the SMC1/3 coils are also much more highly conserved than the SMC2/4 coils when comparing *H. sapiens* to other vertebrates. It is only when *H. sapiens* SMC1/3 are compared to invertebrates such as sea urchin, sea squirt, sea anemone, *Drosophila*, and *C. elegans* that high sequence divergences are observed. Nevertheless, these invertebrate SMC1/3 coils are still more conserved than SMC2/4 or Ndc80/Nuf2p coils. This constraint upon SMC1 coils in animals does not include SMC1β, whose coils show divergences consistent with a spacer rod function. This suggests that the special mechanism involving the coiled coils of SMC1/3 cohesin is found across the entire animal kingdom but is not associated with meiotic cohesin function.

**Table 3:** Animal Tropomyosin, Ndc80, Nuf2p, and SMC Protein and Coil Divergences*.

Animals								
% AA Divergence vs. *H. sapiens*								
	α-Tm	Ndc80	Nuf2p	SMC2	SMC4	SMC1	SMC3	SMC1β
*M. musculus*	0.4	19.0 (16.2)	27.4 (27.9)	10.5 (7.9)	13.3 (10.5)	0.5 (0.6)	0.0 (0.1)	18.9 (16.4)
*R. norvegicus*	0.8p	16.1 (14.8)	21.5 (20.8)	9.6 (6.7)	13.6 (10.5)	0.3 (0.4)	0.6 (0.6)	25.7 (16.2)
*B. taurus*	0.7	8.0 (11.1)	12.2 (8.5)	NA	NA	0.0 (0.2)	0.0 (1.5)	14.2 (10.8p)
*G. gallus*	4.2	27.8 (28.7)	40.2 (46.1)	28.9 (32.9)	28.9 (23.0)	4.3 (5.4)	0.6 (0.7)	44.4 (36.8)
*X. laevis*	6.0	44.8 (43.0)	55.9 (55.3)	32.7 (18.6)	32.7 (23.0)	5.9 (5.9)	4.8 (3.3)	NA
*D. rerio*	7.7	57.3 (54.2)	70.3 (65.3)	29.9 (25.0)	40.5 (31.3)	11.2 (10.8)	4.3 (4.8)	61.1 (46.3)
*S. purpuratus*	70.4p	76.6 (72.6)	NA	NA	60.3 (50.6)	45.4 (37.2)	42.5 (39.2p)	ND
*C. intestinalis*	NA	NA	NA	61.8 (47.3)	65.6 (53.5)	49.4 (43.9)	56.9 (44.7)	ND
*N. vectensis*	60.4	73.4 (68.2)	NA	NA	59.6 (45.7)	55.1 (46.0)	58.5 (50.7)	ND
*D. melanogaster*	51.6	74.8 (82.7)	77.1 (82.2)	77.7 (58.9)	76.9 (60.2)	57.5 (50.3)	60.1 (48.1)	ND
*C. elegans*	43.3	71.3 (78.9)	81.2 (80.5)	73.2 (67.1)	78.4 (68.4)	69.6 (58.3)	69.6 (61.1)	ND

Data presented as percentage sequence divergence for the combined coiled-coils (and in parentheses the divergence for the whole protein).

Tropomyosin sequences are all coiled-coil.

NA = sequence not available.

p = sequence is partial (see Methods).

ND = not determined.

* Portions of data taken from White and Erickson, *Journal of Structural Biology* 154 (2006): 111–121.

### Analysis of SMC1/SMC3 Coiled Coil Mutations in Cornelia de Lange Syndrome

Most CdLS mutations are not in the SMC1/3, but in the accessory protein NIPBL (Nipped-B-Like) [57]. This protein is not a part of the cohesin complex but is involved in loading the cohesin complex onto chromosomes. As noted in the Introduction, however, recent studies have demonstrated that mutations in the coiled-coil domains of both SMC1 and SMC3 can cause CdLS as well [24], [26]. Deardorff et al. [24] reported that the COILS program predicts that the mutations found in the coiled-coil of SMC1 change the probability of the formation of the coiled-coil domains over localized segments of the protein. We repeated this analysis in detail, and in Table 4 summarize the COILS prediction regarding the potential impact of each of these SMC1/3 mutations. The majority (6/7) reduce the probability of coiled-coil formation over relatively small segments. Two mutations: R711W and D831E-Q832Del are predicted to have the greatest impact. The R711W mutation reduces the probability of coiled-coil formation earlier in this segment, and COILS also predicts a shifting of the end of this segment of the coil by 15 amino acids towards the N-terminus of the protein. The D831E-Q832Del mutation would shift the helical groove over the remaining 104 amino acids of this segment of the coil, but produced only a small, localized weakening of the coils. One mutation: R790Q is predicted to increase the probability of coiled-coil formation of this segment by reducing the size of a small upstream interruption. In contrast to the SMC1 mutations, for the only SMC3 coil mutation reported for CdLS patients (E488Del), COILS predicts it will not impact the coil negatively. Overall, it seems that the deleterious effects of the mutations may not involve significant alteration of the coiled-coil structure.

**Table 4:** COILS Predictions for SMC1/3 Mutations in Cornelia de Lange Syndrome.

SMC1		
Mutation	Heptad repeat position	COILS prediction
R196H	*a*	Weakens the start of the coil at K177 - A178 to H187
E493A	*g*	Slight weakening of coil D490 - I503 (end of segment)
R496C	*c*	Weakens the coil at Q498 - E502, shortens end of segment by 1 AA
R496H	*c*	Slight weakening of coil Q498 - E502, shortens end of segment by 1 AA
R711W	*g*	Weakens the coil S703 - T724. Coil lost at M710 versus R725
R790Q	*b* (our helical net analysis: *d*)	Reduce length of small upstream interruption in coil by 1 AA. E783 - I784 versus E783 - I784 - G785.
D831E-Q832del	*d* (helical net analysis: *a*)	Weakening of coil I818 - L821, shifts helical groove through end of this segment at Q936
**SMC3**		
E488Del	*b*	Eliminates slight disruption of the coil at W473. Strengthens coil W483 - L505. Length of coil extended by 2 AAs.

## Discussion

As noted in the Introduction, cohesin in yeast has been implicated in transcriptional silencing and in defining the boundaries of the silenced HMR locus [22], [23], but no role for SMC1β/3 in regulating gene expression has been discovered yet. In animals, however, the CdLS mutations and *Drosophila* data are compelling evidence for a post-mitotic function for SMC1/3 cohesin in the nervous system. In *Drosophila*, SMC1 and Nipped-B are co-localized at multiple locations on the chromosomes, primarily at the promoter regions of active genes [58]. Curiously, reduced levels of Nipped-B reduced expression of the *cut* gene (a homeobox protein important in morphogenesis), while reduced SMC1 increased its expression [25], [59], [60].

Furthermore, the role of cohesin in gene regulation may not be limited to neurons and may not always require an intact cohesin complex. Ghiselli and Iozzo found that overexpressing SMC3 alone approximately 3-fold in NIH and Balb/c 3T3 cells caused them to adopt a transformed phenotype. They also found that SMC3 (SMC1 was not examined) was elevated in 70% of human colon carcinoma samples [61]. Human 293 cells stably transformed by SMC3 overexpression upregulated the expression of at least 65 genes [62]. Though the mechanism by which SMC3 overexpression causes these changes remains unknown, the data suggest further that SMC3 can modulate gene expression without being part of the cohesin complex. It would be interesting to test this for SMC1, and for the isolated coiled-coil segments of the two subunits.

Cohesin's role in regulating gene expression in post-mitotic cells has only recently been added to its list of biological functions. In neurons, the mechanism apparently involves the whole cohesin complex: cleavage of the Rad21 subunit in post-mitotic neurons causes severe defects in axon pruning and larval locomotion [29], and most CdLS defects are due to mutations in the loading factor Nipped-B [57]. In addition, SMC3 appears to have a gene regulatory function on its own, and our results in Table 3 show that SMC3's coiled coils are slightly more constrained than SMC1's from humans to zebrafish. Our work now suggests that the high conservation of the coiled coils of cohesin across the animal kingdom may be an important part of these mechanisms. It is interesting that five out of seven CdLS mutations in SMC1 change positively charged arginines to neutral residues. These arginines may be involved in binding to the negatively charged phosphates of DNA.

### Conclusions

Our previous analysis showed that the coiled coils of cohesin are very highly conserved across vertebrates, which implied a function involving the entire length and circumference of the coiled coil. At that time we suggested that the surfaces of the coils would be involved in sister chromatid cohesion, which was the only known function for cohesin. A recent study has provided evidence for lateral interactions of yeast cohesins during that process, presumably along the length of their coiled coils [63]. This mechanism is apparently not one that requires extreme conservation of the coiled coil sequence, since we found that the coils are not highly conserved in Saccharomycotina. However, several recent studies have demonstrated that cohesin also has a separate function involving regulation of gene expression in post-mitotic neurons and other cells. We now suggest that the coiled coils of cohesin may play a key role in this second mechanism. The high conservation of the coiled coils of cohesin in metazoans supports the hypothesis that the entire surface of the coils may be involved in binding interactions, perhaps to the DNA of the various genes they regulate.
